# Preparation of quinoa bran dietary fiber-based zinc complex and investigation of its antioxidant capacity *in vitro*

**DOI:** 10.3389/fnut.2023.1183501

**Published:** 2023-05-25

**Authors:** Chunhong Wei, Xinhui Wang, Xiujie Jiang, LongKui Cao

**Affiliations:** ^1^College of Food Science, Heilongjiang Bayi Agricultural University, Daqing, China; ^2^National Coarse Cereals Engineering Research Center, Heilongjiang Bayi Agricultural University, Daqing, China

**Keywords:** quinoa bran, soluble dietary fiber (SDF), zinc sulfate heptahydrate, antioxidant, complex

## Abstract

In order to improve the economic utilization of quinoa bran and develop a safe and highly available zinc ion biological supplement. In this study, a four-factor, three-level response surface optimization of quinoa bran soluble dietary fiber (SDF) complexation of zinc was studied. The effect used four factors on the chelation rate was investigated: (A) mass ratio of SDF to ZnSO_4_.7H_2_O, (B) chelation temperature, (C) chelation time, and (D) pH. Based on the results of the single-factor test, the four-factor three-level response surface method was used to optimize the reaction conditions. The optimal reaction conditions were observed as mentioned here: the mass ratio of quinoa bran SDF to ZnSO_4_.7H_2_O was 1, the reaction temperature was 65°C, the reaction time was 120 min, and the pH of the reaction system was 8.0. The average chelation rate was 25.18%, and zinc content is 465.2 μg/g under optimal conditions. The hydration method rendered a fluffy quinoa bran SDF structure. The intramolecular functional groups were less stable which made the formation of the lone pairs of electrons feasible to complex with the added divalent zinc ions to form a quinoa bran soluble dietary fiber-zinc complex [SDF-Zn(II)]. The SDF-Zn(II) chelate had higher 2,2-diphenylpicrylhydrazyl (DPPH), ABTS^+^, hydroxyl radical scavenging ability, and total antioxidant capacity. Therefore, metal ion chelation in dietary fiber is of biological importance.

## 1. Introduction

Zn is one of the most common metal elements in the human body and plays an important role as a signaling factor in growth and development (1). It is an important component and is a signaling factor of enzymes and proteins. It also acts as an essential and prevalent ionic signal in numerous cells and tissues (2, 3). It is known as the “life element” in the human body (4). The World Health Organization has reported Zn deficiency as a serious problem. Zn deficiency in pregnant women during lactation and in children during early childhood may delay growth, induce a loss of appetite, low immunity and reproductive capacity, and various types of cancer (5, 6). The bioavailability of inorganic zinc salts in first-generation zinc supplements is very low, and their byproducts can cause severe gastrointestinal irritation. Although second-generation zinc supplements significantly reduce gastrointestinal irritation, long-term use can result in calcium deficiency and iron deficiency anemia. Third-generation zinc supplements are bio-based zinc (7) that are non-toxic and harmless but only contain Zn. Therefore, it is necessary to develop new bio-based zinc supplements with high bioavailability and antioxidant activities. Dietary fiber (DF), a nutrient required by the human body, is defined as the seventh macronutrient by nutrition and is classified as soluble DF (SDF) and insoluble DF (IDF) based on its hydrolytic properties. Relevant studies have shown that the physiological functions of different types of DF depend largely on their physicochemical properties, one of which is solubility. SDF is more readily accessible and metabolized by fiber-degrading microorganisms in the intestine as compared to IDF to produce a series of beneficial functional metabolites. At present, there is almost no study on the physical synthesis of SDF by introducing transition metal ions. Reports on metal ion complexation within polysaccharides have been widely studied, and the structure and properties of water-soluble DF are similar to those of polysaccharides. Therefore, this study was conducted with reference to the molecular synthesis and structural reorganization of polysaccharides to endow them with new activities. It was found that the structural synthesis of polysaccharides could retain their biological activities, and metal elements may have synergistic effects in the production of new biological functions (8). Chen et al. (9) extracted garlic polysaccharides and prepared iron complexes with strong antioxidant properties. In summary, SDF can be used as ligands for complexation with transition metals to form new complexes.

According to the Food and Agriculture Organization (FAO) of the United Nations, quinoa, a single plant that can meet the basic nutritional needs of the human body, has significant potential for alleviating hunger and partially solving the global food crisis in the future. It is rich in dietary fiber, protein, minerals, vitamins, and many phytochemical elements has a positive effect on maintaining and strengthening health by preventing diseases (10). Quinoa bran, a major high-value byproduct, is mainly used in the animal feed industry resulting in a large waste of economic resources. Therefore, in this study, we used quinoa bran soluble dietary fiber (SDF) as a carrier and introduced Zn ions *via* microwave chelation to prepare SDF-Zn(II). The structural characterization and bioactivity assessment of SDF and SDF-Zn(II) complexes were performed. This research not only increases the economic utilization of quinoa bran, but also develops a safe and high-utilization zinc ion biological supplement, which has high antioxidant activity *in vitro*.

## 2. Materials and methods

### 2.1. Materials and reagents

Quinoa bran (variety: gerbera; origin: Qinghai) was stored at 4°C for use. Petroleum ether, hydrochloric acid, sodium hydroxide, and zinc sulfate heptahydrate were procured from Tianjin Damao Chemical Reagent Factory. Citric acid was purchased from Beijing Beihua Fine Chemicals Co, Ltd. All other reagents were of analytical grade.

### 2.2. Determination of routine indicators of quinoa bran

Determination of fat content (GB/T5009.6-2016); moisture content (GB/T5009.3-2016); protein content (GB/T5009.5-2016); ash content (GB/T5009.4-2016); starch content (GB/T5009.9-2016); and fiber content (AACC32-07) (11).

### 2.3. The synthesis of soluble dietary fiber from quinoa bran

#### 2.3.1. Extraction of dietary fiber from quinoa bran

Quinoa bran was dried at 40°C to maintain the moisture content in the range of 8–9%, ultra-micronized using a vibratory mill with a single feeding volume of 2000 g and a crushing time of 60 min, passed through an 80 mesh sieve, and sealed at 4°C for storage. Following ultra-micronization, the quinoa bran powder was defatted for 12 h using petroleum ether and was stored. The enzymatic extraction of quinoa bran SDF was performed as described here: 50 g of pretreated quinoa bran was weighed followed by the addition of two 500 mL of phosphate buffer solutions at a mass ratio of 1:50 (g/g). pH was maintained at 6.0 using 0.01 mol L^–1^ hydrochloric acid followed by the addition of 100 μL of high-temperature resistant α-amylase. The reaction was carried out in a water bath at 95–100°C for 25 min. A total of 1000 μL of neutral protease solution was added by maintaining the pH = 7, and the reaction was carried out at 60°C for 30 min. Again, 1000 μL of amyloglucosidase was added at pH = 4.5, and the reaction was carried out at 60°C for 30 min until it failed to turn blue in the presence of iodine, followed by the inactivation of the enzyme (>100°C, 10 min). The enzymatic solution was filtered, and the residue was washed and freeze-dried to obtain quinoa bran IDF, the filtrate was centrifuged (4000 rpm, 20 min), and concentrated to 30% of the original volume. The alcoholic sedimentation was incubated overnight with four times the volume of 95% ethanol. The supernatant was centrifuged at 4000 rpm for 25 min. The resulting precipitate was freeze-dried to obtain quinoa bran SDF (12, 13).

#### 2.3.2. Purification of quinoa bran soluble dietary fiber

Quinoa bran soluble dietary fiber was accurately weighed (0.5 g), dissolved in distilled water, and the volume was made to 100 mL. The protein was removed using the Sevag method. The Sevag reagent was prepared in a ratio of n-butanol: trichloromethane (1:5). The pH of quinoa bran SDF solution was maintained at 8.5 with ammonia. A total of 50 mL of 30% H_2_O_2_ was added and decolorized by heating at 40°C for 60 min. The organic reagents were removed by using a rotary evaporator and dialyzed for 48 h. The dialysate was concentrated to a certain volume and precipitated with anhydrous ethanol. The precipitate was placed overnight in a refrigerator at 4°C. The precipitate was centrifuged at 9,000 r/min for 5 min the following day and washed with anhydrous ethanol, acetone, and ether. The washed precipitate was freeze-dried to obtain the purified quinoa bran soluble dietary fiber (12).

#### 2.3.3. Preparation of soluble dietary fiber-zinc chelate from quinoa bran

The preparation of SDF-Zn(II) chelate was conducted by referring to the method described by Zhao et al. (14–16). A total of 10.00 g of quinoa bran SDF was accurately weighed and dissolved in 500 mL of distilled water, followed by stirring for 20 min at 70°C. The quinoa bran SDF solution was added to an equal volume of ZnSO_4_.7H_2_O solution at a concentration of 2–6 g L^–1^. The reaction was stirred at 50–70°C in a water bath, reaction time was 90–150 min. After cooling to room temperature and dialyzing for 12 h, the supernatant was concentrated to one-fourth of its original volume. Alcoholic sedimentation was performed by adding four times the volume of 95% ethanol for 12 h. The SDF-Zn(II) chelate was obtained by centrifugation and freeze-drying.

#### 2.3.4. SDF-Zn(II) chelation process single factor analysis

The effects of four factors, including chelation temperature, chelation time, solution pH, and the mass ratio of SDF to ZnSO4.7H_2_O. Single-factor tests were conducted by varying the following parameters: (i) the mass ratio of SDF to ZnSO_4_.7H_2_O (1:0.4, 1:0.6, 1:0.8, 1:1, and 1:1.2); (ii) chelation temperature (40, 50, 60, 70, and 80°C); (iii) chelation time (30, 60, 90, 120, and 150 min); and (iv) solution pH (5, 6, 7, 8, and 9).

#### 2.3.5. SDF-Zn(II) chelation process response surface analysis

A response surface optimization design was developed for the chelation rate of SDF-Zn(II) with quinoa bran as the response value based on the univariate experiment. The Design-Expert 10.0.3 central combination experimental design was used to examine the (i) mass ratio of ZnSO_4_.7H_2_O to SDF, (ii) chelation temperature, (iii) chelation time, and (iv) pH as independent variables to establish the experimental model. The response surface experimental design factors and their levels are listed in Table 1.

**TABLE 1:** Box–Behnken test factors and levels.

Level	Factors			
	**A**	**B**	**C**	**D**
	**The mass ratio of SDF to ZnSO_4_-7H_2_O**	**Chelation temperature (°C)**	**Chelation time (min)**	**pH**
−1	1:0.6	50	90	7
0	1:0.8	60	120	8
1	1:1.0	70	150	9

#### 2.3.6. SDF-Zn(II) chelation rate analysis

A total of 0.5 g of SDF-Zn(II) chelate was weighed accurately followed by the addition of 50 mL of distilled water to dissolve the sample. A total of 3 drops of dimethoate orange were added as an indicator. The solution was titrated with 0.02 mol L^–1^ ethylenediamine tetraacetic acid (EDTA) standard solution until the color of the solution changes from purple red to a bright yellow, considered as the endpoint of the titration. Another accurately weighed 0.5 g of undialyzed chelate was dissolved in 50 mL of distilled water followed by the addition of three drops of dimethoate orange as an indicator. The solution was titrated with 0.02 mol L^–1^ EDTA standard solution until it turns from purple-red to bright yellow (17, 18). The chelation rate was calculated using Equation 1.


Chelationrate(%)

(1) $$=\frac{C\,V_{1}}{C\,V_{2}}\times 100\%\,\ldots \,\ldots \,\ldots \,\ldots \,\ldots \,\ldots \,\ldots \,\ldots \,\ldots ..$$

Where C: EDTA solution concentration mol L^–1^.

V_1_: Volume of EDTA solution consumed during the titration of the chelated trace elements, mL.

V_2_: Volume of EDTA solution consumed during the titration of total trace elements, mL.

#### 2.3.7. SDF-Zn(II) chelation content analysis

Soluble dietary fiber-Zn(II) was diluted 1:9 with 4-(1,1,3,3-tetramethylbutyl)phenyl-polyethylene glycol (Triton-X-100), acidified with 2 mmol/L hydrochloric acid, and ashed with 20 mL nitric acid + perchloric acid + sulfuric acid (ratio 10:6:4). The sample loading volume was 100 L, which was measured with an iCETM 3400 AAS atomic absorption spectrometer and used to calculate the zinc content for subsequent experiments.

### 2.4. Analysis of soluble dietary fiber structure of quinoa bran before and after synthesis

#### 2.4.1. Scanning electron microscopy (SEM) analysis

Morphological and microstructural studies of SDF and SDF-Zn(II) samples were conducted by SEM. The samples were placed on double-sided adhesive tape and coated with a thin gold layer. Images were acquired at an accelerating voltage of 50.0 kV. The micrographs were magnified 1000 × and recorded (19).

#### 2.4.2. Fourier transform infrared (FTIR) spectroscopy analysis

Soluble dietary fiber and SDF-Zn(II) (2 mg) were mixed with 200 mg of powdered potassium bromide and pressed into tablets for analysis *via* FTIR spectroscopy at 4,000–400 cm^–1^ (20).

### 2.5. Analysis of *in vitro* antioxidant properties of SDF and SDF-Zn(II)

#### 2.5.1. Analysis of DPPH free radical scavenging rate

2,2-diphenylpicrylhydrazyl (DPPH) radical scavenging ability was determined using the method described by Yang et al. (21) with appropriate modifications. A 0.2 mmol L^–1^ DPPH-ethanol solution was prepared, and the samples were diluted with deionized water to 1, 2, 3, 4, 5, and 6 mg mL^–1^. The VC solution was used as a positive control. The samples were incubated for 30 min at 37°C after mixing according to the formula. The absorbance was measured at 517 nm using distilled water as the blank control. Each sample was measured three times in parallel using Equation 2.


DPPHfreeradicalscavengingrate(%)

(2) $$=\left[1-\frac{A_{1}-A_{2}}{A_{0}}\right]\times 100\%\,\ldots \,\ldots \,\ldots ..$$

Where A_1_ = 1 mL sample + 2 mL DPPH, A_2_ = 1 mL sample + 2 mL methanol, and A_0_ = 1 mL methanol + 2 mL DPPH.

#### 2.5.2. 2,2′-Azino-bis (3-ethylbenzthiazoline-6-sulfonic acid (ABTS^+^)) free radical scavenging capacity analysis

The free radical scavenging rates of quinoa bran SDF and SDF-Zn(II) were determined according to the method described by Yang et al. (21) with minor adjustments. Distilled water was used to prepare 0.5, 1, 1.5, 2, and 2.5 mg mL^–1^ sample solutions. A total of 7 mol L^–1^ ABST solution was mixed with 2.45 mol L^–1^ potassium persulfate solution at a 1:1 ratio by volume to form a buffer solution. The mixture was allowed to react for 16 h at 37°C in dark to form the ABTS stock solution. The ABTS stock solution was diluted with phosphate buffer at pH 7.4 to an absorbance value of 0.700 ± 0.05 at 734 nm and was used as the working solution. The working solution was mixed with different concentrations of quinoa bran SDF solutions and incubated for 6 min in the dark. The absorbance was measured at 734 nm. Each group was tested in triplicate: distilled water instead of the sample was used as the blank control group, and distilled water instead of the reagent was used as the reagent control group. The radical scavenging rate was calculated using Equation 3.


$$A\,B\,S\,T^{+}\,F\,r\,e\,e\,r\,a\,d\,i\,c\,a\,l\,s\,c\,a\,v\,e\,n\,g\,i\,n\,g\,r\,a\,t\,e\%$$

(3) $$=\left[1-\frac{A_{1}-A_{2}}{A_{0}}\right]\times 100\,\text{…………}.$$

Where: A_1_ = 0.2 mL sample dilution + 1.2 mL ABTS + diluent; A_2_ = 0.2 mL sample dilution + 1.2 mL anhydrous methanol; and A_0_ = 0.2 mL anhydrous methanol + 1.2 mL ABTS + diluent.

#### 2.5.3. Analysis of hydroxyl radical scavenging capacity

Hydroxyl radicals are generated according to the reaction Fe^2+^ + H_2_O_2_ = Fe^3+^OH^–^ + OH^–^ (22). The ability of the samples to scavenge hydroxyl radicals was determined by referring to the method described by Benton (23) with appropriate modifications. The sample was weighed and prepared with concentrations of 1, 2, 3, 4, 5, and 6 mg mL^–1^. The VC solution was used as a positive control. A total of 1 mL of 9 mmol L^–1^ FeSO_4_ solution, 1.0 mL of H_2_O_2_ solution (0.5%, v/v), and 1 mL of 9 mmol L^–1^ salicylic acid solution were added to 1.0 mL of the sample solution. The mixture was allowed to react at 37°C for 30 min. Absorbance was measured at 510 nm, and each sample was measured three times in parallel. The hydroxyl radical scavenging rate was calculated using Equation 4:


H⁢y⁢d⁢r⁢o⁢x⁢y⁢l⁢r⁢a⁢d⁢i⁢c⁢a⁢l⁢s⁢c⁢a⁢v⁢e⁢n⁢g⁢i⁢n⁢g⁢r⁢a⁢t⁢e%

(4) $$=\frac{A_{0}-A_{1}}{A_{0}}\times 100\%\,\ldots \,\ldots \,\ldots \,\ldots \,\ldots \,\ldots \,\ldots \,\ldots \,\ldots \,\ldots ..$$

Where A_0_ = the absorbance of the blank control at 510 nm and A_1_ = the absorbance of the sample to be measured at 510 nm.

#### 2.5.4. Total antioxidant capacity analysis

The total antioxidant capacity of the samples was determined using the ferric ion reducing capacity method following the method described by Ridlon et al. (24) with appropriate modifications. The FRAP solution was prepared from an acetate buffer solution (pH 3.6, 300 mM), 10 mM TPTZ dissolved in 40 mM hydrochloric acid solution, and 20 mM ferric chloride solution at 10:1:1 (v/v). The samples were diluted to 1, 2, 3, 4, 5, and 6 mg mL^–1^ with deionized water, and 100 μL of the sample solution was added to 5 mL FRAP. The mixture was heated for 20 min in a water bath at 37°C under stirring, and the absorbance was measured at 593 nm. Each sample was repeated three times. The concentration range of 0.2–0.8 mmol L^–1^ FeSO_4_ standard solution was prepared, and a standard curve was plotted to reflect the total antioxidant capacity of the samples by FeSO_4_ equivalent concentration.

### 2.6. Data processing

Data were statistically analyzed using the statistical software SPSS 17.0. One-way ANOVA was used to compare differences between groups, and the Tukey’s test was used for data that met the chi-square test. The Tamhane test was used for data that did not meet the chi-square test. Statistical significance was set at *P* < 0.05 for indicating that the difference between groups was statistically significant. Significant differences between groups were marked as *P < 0.05, ^**^P < 0.01, ^***^P < 0.001, and data were expressed as the mean ± standard error.

## 3. Results

### 3.1. Analysis of the basic nutritional composition of SDF and SDF-Zn(II)

As shown in Table 2: quinoa bran was rich in starch, protein, and total dietary fiber. The total DF content of quinoa bran was 27.28%, higher than the fiber content of quinoa seeds reported by Marcin et al. (25) indicating a significant advantage of using quinoa bran for DF extraction. Starch and fat are not detected in SDF or SDF-Zn(II) (II). This demonstrates that the enzyme method extracts the quinoa bran SDF with greater purity, making it suitable for future experiments.

**TABLE 2:** Main components of quinoa bran.

Component (%)	Starch	Fat	Protein	Moisture	Ash	Total dietary fiber
Quinoa bran	16.92 ± 0.25	8.01 ± 0.06	22.17 ± 0.62	9.45 ± 0.35	16.17 ± 0.44	27.28 ± 1.56
SDF	/	/	5.03 ± 0.22	4.72 ± 0.11	2.38 ± 0.07	80.17 ± 0.21
SDF-Zn(II) (II)	/	/	4.37 ± 0.33	4.03 ± 0.04	2.61 ± 0.05	81.62 ± 0.37

### 3.2. Analysis of process conditions for chelation synthesis of quinoa bran SDF-Zn(II)

#### 3.2.1. Single-factor experimental analysis

**FIGURE 1:**
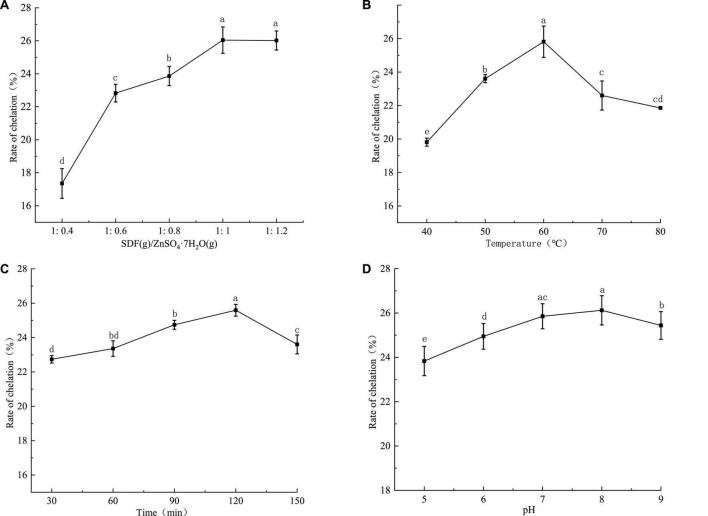
Single factor test results of SDF-Zn(II) (II) chelation synthesis of quinoa bran. In **(A)** mass ratio of quinoa soluble dietary fiber to ZnSO4-7H_2_O, **(B)** chelation temperature, **(C)** chelation time, and **(D)** pH; different letters in a, b, c, and d indicate significant differences between chelation rates (*P* < 0.05).

The results of the single-factor test showed that the optimal conditions for the synthesis of SDF-Zn(II) were: the mass ratio of SDF to ZnSO_4_-7H_2_O was 1, the reaction temperature was 60°C, the reaction time was 120 min, and the pH = 8 (Figure 1).

#### 3.2.2. Box–Behnken test analysis

The four-factor for the three-level response surface design scheme are as follows: (A) mass ratio of SDF to ZnSO_4_.7H_2_O, (B) chelation temperature, (C) chelation time, and (D) pH. The values are shown in Table 3.

**TABLE 3:** Response surface design and results.

Serial number	Factors				Chelation rate (%)
	**A**	**B**	**C**	**D**	
1	1	1	0	0	25.92 ± 0.91
2	0	−1	0	−1	23.46 ± 1.22
3	0	1	0	1	23.50 ± 0.99
4	−1	0	1	0	22.69 ± 1.16
5	1	0	0	−1	23.44 ± 1.87
6	0	−1	0	1	23.67 ± 1.37
7	−1	1	0	0	22.74 ± 1.07
8	−1	0	0	−1	22.76 ± 1.26
9	1	0	0	1	25.39 ± 1.09
10	1	−1	0	0	24.18 ± 1.23
11	0	0	0	0	24.97 ± 0.121
12	1	0	1	0	25.35 ± 1.07
13	1	0	−1	0	25.18 ± 0.97
14	0	0	0	0	24.38 ± 1.04
15	0	0	1	1	24.88 ± 1.02
16	−1	−1	0	0	22.18 ± 1.15
17	−1	0	−1	0	22.22 ± 0.17
18	0	0	−1	−1	23.78 ± 0.89
19	0	0	1	−1	23.85 ± 0.24
20	0	1	0	−1	24.02 ± 0.86
21	−1	0	0	1	22.26 ± 0.65
22	0	0	−1	1	23.33 ± 0.29
23	0	−1	1	0	23.32 ± 0.48
24	0	1	1	0	24.73 ± 0.85
25	0	−1	−1	0	23.31 ± 0.12
26	0	0	0	0	24.59 ± 0.56
27	0	0	0	0	24.45 ± 0.68
28	0	1	−1	0	24.67 ± 0.86
29	0	0	0	0	24.51 ± 0.59

#### 3.2.3. Regression equation significance analysis

The experimental data were analyzed using Design-Expert 10.0.3 software to obtain the quadratic multinomial regression equation as shown in Equation 5:


(5) Chelationrate(%)=24.58+1.22×A+0.46×B-0.19

×C+0.14×D+0.30×A⁢B-0.075×A⁢C+0.61

×A⁢D+0.012×B⁢C-0.18×B⁢D+0.37×C⁢D-0.54

×A⁢2-0.36×B⁢2-0.16×C⁢2-0.53×D⁢2⁢…⁢…⁢…⁢…⁢…⁢….

According to the results shown in Table 4, the optimal chelation conditions under the joint influence of the four factors are as shown here: *A* = 1:0.98, *B* = 65.13°C, *C* = 120.17 min, and *D* = 8.186. The model predicted a chelation rate of 25.92% under these conditions.

**TABLE 4:** Analysis of variance in response surface regression model.

Source	Square and	Degree of freedom	Mean square	*F*-value	*P*-value	Significance
Models	26.90	14	1.92	12.14	<0.0001	**
A	17.79	1	17.79	112.42	<0.0001	**
B	2.48	1	2.48	15.70	0.0014	**
C	0.45	1	0.45	2.86	0.1130	
D	0.25	1	0.25	1.56	0.2324	
AB	0.35	1	0.35	2.20	0.1602	
AC	0.022	1	0.022	0.14	0.7118	
AD	1.50	1	1.50	9.48	0.0082	**
BC	6.250E-004	1	6.250E-004	3.950E-003	0.9508	
BD	0.13	1	0.13	0.84	0.3744	
CD	0.55	1	0.55	3.46	0.0840	
A2	1.86	1	1.86	11.77	0.0041	**
B2	0.85	1	0.85	5.37	0.0361	*
C2	0.17	1	0.17	1.06	0.3206	
D2	1.84	1	1.84	11.61	0.0043	**
Residuals	2.22	14	0.16			
Loss of proposed items	2.00	10	0.20	3.74	0.1077	
Pure error	0.21	4	0.054			
Total sum	29.11	28				

*Indicates significance (*P* < 0.05). **Indicates high significance (*P* < 0.01).

#### 3.2.4. Optimal process conditions experimental verification analysis

The quinoa bran SDF chelation process was modified by combining the feasibility of the actual process setup based on the results predicted by the software. The validation test was conducted with the following parameters of the reaction system: *A* = 1, *B* = 65°C, *C* = 120 min, and *D* = 8.0. The relative error between the actual value and the predicted value (25.92%) is 0.74%. This indicated that actual values are in good agreement with the predicted values of the model. Thus, the regression model fit is high and operable.

### 3.3. Analysis of soluble dietary fiber structure of quinoa bran before and after synthesis

#### 3.3.1. SEM analysis

**FIGURE 2:**
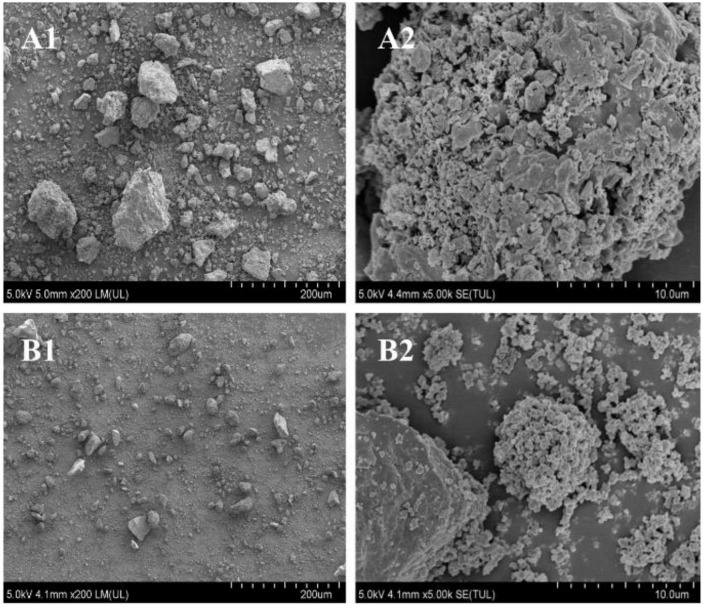
Scanning electron microscopy (SEM) images of SDF and SDF-Zn(II). **(A1)** Quinoa bran SDF (× 200); **(A2)** quinoa bran SDF (× 5000); **(B1)** SDF-Zn(II) (× 200); **(B2)** SDF-Zn(II) (× 5000).

The SEMS images of SDF and SDF-Zn(II) are shown in Figure 2. The image of SDF shows loosely agglomerated and aggregated large particles; SDF-Zn(II) with more loose and homogeneous pores.

#### 3.3.2. FTIR spectroscopy

**FIGURE 3:**
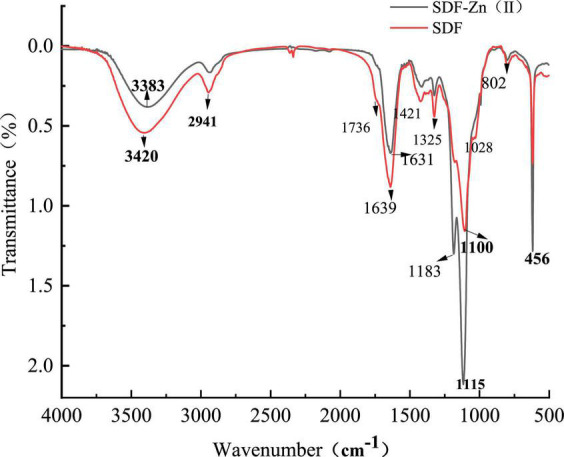
Original Infrared scan of SDF and SDF-Zn(II) complex.

Soluble dietary fiber and SDF-Zn(II) exhibit similar IR spectral profiles as shown in Figure 3, indicating that the synthesis did not significantly affect their chemical structures.

### 3.4. Analysis of *in vitro* antioxidant properties of SDF and SDF-Zn(II)

**FIGURE 4:**
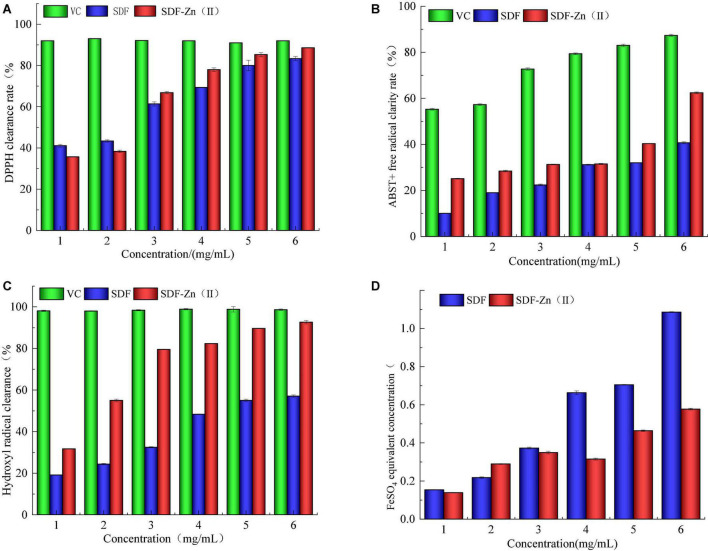
Analysis of *in vitro* antioxidant properties of SDF and SDF-Zn(II). **(A)** DPPH clearance rate; **(B)** ABTS + free radical clarity rate; **(C)** Hydroxyl radical clearance; and **(D)** FeSO_4_ equivalent concentration.

As shown in Figure 4, DPPH, ABTS^+^, hydroxyl radical scavenging ability, and the total antioxidant capacity of SDF-Zn(II) are complex significantly higher than those of SDF and lower than those of VC.

## 4. Discussion

The chelation rate of quinoa bran SDF-Zn(II) increases with the increasing mass ratio (1:0.4–1:12) of quinoa bran SDF to ZnSO_4_.7H_2_O as shown in Figure 1A. The chelation rate is the maximum (26.04%) and reaches the equilibrium at the ratio of 1:1. The binding sites of zinc ions in the solution are less when the quinoa bran SDF content is low. This reduces the probability of chelation, resulting in a low chelation rate. The number of binding sites increases with an increase in the quinoa bran SDF content which increases the chelation rate. Intermolecular forces and increased solution viscosity induce the encapsulation of binding sites after a particular quinoa bran SDF concentration limit; this decreases the chelation rate (16). As shown in Figure 1B, the temperature had a significant effect on the chelation rate. The chelation rate of SDF-Zn(II) increased with the increase of the reaction temperature between 40 and 60°C. The maximum chelation rate (25.81%) is observed at the chelation temperature of 60°C. The rate of molecular motion increases with temperature rise. The molecular energy and the frequency of intermolecular collisions also increase with the increase of the reaction temperature. This increases the collision probability between Zn ions and SDF chelation sites, thus increasing the chelation rate. Since the heat of the system is not released, the intermolecular collisions are too intense at temperatures >60°C. Thus, products are decomposed and are not conducive to the reaction (26). The chelation rate increased within 30–120 min as shown in Figure 1C. The chelation rate is reduced over 120 min. The chelating sites of SDF cannot sufficiently interact with zinc ions within a short reaction time (<120 min). Thus, the reaction is incomplete with a low chelation rate. Chelation reaches equilibrium with prolongation of the reaction time. However, a long chelation time (>120 min) might dissociate the chelated metal ions and SDF, thereby decreasing the chelation rate. The chelation rate increases and then decreases with increasing pH as shown in Figure 1D. The maximum chelation rate was 25.6% at pH 8. The high concentration of free OH^–^ ions under alkaline conditions increases the chelation rate with zinc ions. In contrast, the high concentration of H^+^ ions between pH 5 and 7 induces a ligand reaction between the reactive groups in SDF and H^+^. Thus, it competes with zinc ions resulting in a low chelation rate.

An ANOVA was performed on the regression equation to examine the usability of the regression model and the synergistic effect between factors. The results presented in Table 4 indicate the total models of the regression equation reach a highly significant level (*P* < 0.01). Moreover, the misfit term is not significant (*P* > 0.05) indicating the accuracy of the proposed model. The equation is consistent with the experimental results. It is reliable and can predict the effect of A, B, C, and D on the chelation rate of Zn with quinoa bran SDF. The order of the effect of these factors on the chelation rate is determined based on the absolute values of the primary term coefficients in the binary regression equation: A > B > C > D. The coefficient of determination (R^2^) of the model is 0.9239 establishing the high significance of the model. *R*^2^adj = 0.9860 explains 95.11% of experimental response value variation and is close to the predicted correlation coefficient *R*^2^. Thus, this experimental model fits well with real data and has practical significance. The model can be used to analyze and predict the optimal extraction process of the chelation rate. From the *F*-values in the table, the primary term coefficients SDF to ZnSO4.7H_2_O mass ratio (A), chelation temperature (B), and pH (D) had significant effects on dietary fiber zinc chelate, and the chelation time (C) had effects on chelation rate. The maximum chelation rate is used as the optimization target to synergistically consider the interaction between the factors on the chelation rate and further determine the global optimal combination. According to the results of Design-Expert 10.0.3 software, optimal chelation conditions under the joint influence of the four factors are as shown here: *A* = 1:0.98, *B* = 65.13°C, *C* = 120.17 min, and *D* = 8.18. The model predicted a chelation rate of 25.92% under these conditions.

Scanning electron microscopy was used to analyze the changes in the surface microstructures of the samples. In this study, SDF-Zn(II) complexes were prepared by the chelation reaction of quinoa bran SDF with zinc sulfate. The SEMS images of SDF and SDF-Zn(II) are shown in Figure 2. The image of SDF at 200 × magnification show loosely agglomerated and aggregated large particles; the same magnification of SDF-Zn(II) in Figure 2B1 shows more homogeneous and loose structure with fewer agglomerated lumps. Figure 2B2 shows SDF-Zn(II) with more loose and homogeneous pores. This might be due to the increase in temperature and pressure during chelation synthesis. The SDF structure is disrupted to form small surface particles and increased surface area. Thus, more reactive groups are exposed which are closely related to their physicochemical and functional properties (27, 28). SDF comprises glucose as the main molecular skeleton to form a macromolecular structure *via* hydrogen bonds. The glycosidic bond breaks partially during chelation with Zn contributing to a more homogeneous SDF structure, increased surface area, and formation of loose pores (29, 30).

As shown in Figure 3, the synthesis did not significantly affect the chemical structures of SDF. The broad absorption peaks near 3420 and 3383 cm^–1^ are attributed to the hydroxyl stretching vibrations of the hydroxyl groups of cellulose and hemicellulose (31). The spectrum shows a shift of the 3420 cm^–1^ peak to 3383 cm^–1^ and a narrowing of the (-OH) absorption peak of SDF-Zn(II). Presumably, the hydroxyl group of SDF interacts with divalent zinc ions to form intramolecular hydrogen bonds. In addition, the C-H stretching vibration peaks at 2942 cm^–1^ of methyl and methylene sugars are the characteristic absorption peaks of sugars. The peaks near 1639 and 1631 cm^–1^ may be the characteristic absorption peaks of carbon–oxygen double bonds in glyoxalate, indicating the possible presence of glyoxalate in SDF and SDF-Zn(II). Both SDF and SDF-Zn(II) spectra show the typical functional groups of polysaccharides. The principle of the chelation reaction is similar to that of polysaccharides. *C* = O double bond absorption peaks at 1468 and 1488 cm^–1^ of SDF-Zn(II) shifts to 1488 cm^–1^ indicating the involvement of *C* = O in forming bonds (32, 33). Strong absorption peaks at 1100 and 1028 cm^–1^ indicate the presence of pyranose units in SDF and SDF-Zn(II). The absorption peak at 802 cm^–1^ indicates the α-D-pyranose conformation. The IR spectra of both SDF and SDF-Zn(II) are similar with; only a change in the peak intensity and peak shift. The enhancement of the peaks at 1627 and 1488 cm^–1^ indicates that chelation-based synthesis affects the structure of SDF functional groups (34). However, the absorption band at 3388 cm^–1^ turned weak and showed a shift to a higher wavenumber of 3402 cm^–1^in SDF-Zn(II), indicating that the hydroxyl groups might be involved in coordination with zinc ions (35).

2,2-diphenylpicrylhydrazyl, a crystalline radical, is relatively stable and easily soluble in organic solvents, such as ethanol, forming a dark purple solution. DPPH molecules contain single electrons. When a reducing sample is added to the DPPH molecule, it forms a pairing with the single electron and the dark purple color of the solution fades. The stronger the antioxidant activity of the test sample, the more obvious the fading. Both SD and SDF-Zn(II) exhibit clear DPPH radical-scavenging abilities as shown in Figure 4A. The scavenging rate increases from 25.12 to 31.28% when the mass concentration increases from 1 to 3 mg mL^–1^. ABTS, a diamine salt, can be oxidized by potassium persulfate to generate more stable ABTS + radicals. The aqueous ABTS solution is light green. Dark green ABTS + stock solution was obtained after overnight incubation with potassium persulfate. A sample with antioxidant activity can pair with a single electron in ABTS + to discolor the solution; the higher the antioxidant activity, the lighter the green color of the reaction solution. Both SDF and SDF-Zn(II) exhibit ABTS + radical-scavenging ability as shown in Figure 4B. The ABTS + radical-scavenging ability of 6 mg mL^–1^ SDF-Zn(II) is 88.61%.

## 5. Conclusion

Dietary fiber and metal ions have important physiological effects on the human body. Combining them has important implications in life sciences. (i) The new complexes formed by DF and metal ions have dual physiological functions which improve the healthcare value of DF and metal ions. (ii) The *in vitro* physicochemical property of complexes formed by DF and metal ions is important for the development of new organometallic ion supplements. (iii) Additionally, substances will generate new physiological functions and have better application prospects in the food and medical fields. A zinc gluconate oral solution is a typical example of a good metal complex with metal ion supplementation. Therefore, DF-based metal ion chelates play an important role in life processes, and further studies on their interactions and physiological functions are necessary.

In this study, quinoa bran was used as the raw material. The quinoa bran SDF was extracted, modified, and analyzed for its structure and antioxidant capacity using the national standard method. The results showed that the chelation rate was 25.18% at the mass ratio of SDF to ZnSO_4_.7H_2_O is 1, the reaction temperature is 65°C, and the reaction time is 120 min at pH = 8. The prepared SDF-Zn(II) chelate retained the molecular structure of SDF. However, the chelated complexes had smaller surface particles, increased surface area, and more exposed reactive groups as compared to SDF. SDF-Zn(II) had higher DPPH, ABTS +, hydroxyl radical scavenging ability, and total antioxidant capacity. Our results suggest that SDF-Zn(II) may be an effective antioxidant Zn supplement for use in functional food and pharmacology.

## Data availability statement

The original contributions presented in this study are included in the article/supplementary material, further inquiries can be directed to the corresponding author.

## Author contributions

CW and XW were responsible for the design and overall management of the entire study, analyzed the data, and edited the manuscript. XJ provided validation, formal analysis, writing, review, and editing. LC supervised, wrote, reviewed, edited, and acquired funding. All authors have read and agreed to publish the current version of the manuscript.
